# Influence of nanoparticle addition on the formation and growth of intermetallic compounds (IMCs) in Cu/Sn–Ag–Cu/Cu solder joint during different thermal conditions

**DOI:** 10.1088/1468-6996/16/3/033505

**Published:** 2015-05-20

**Authors:** Ai Ting Tan, Ai Wen Tan, Farazila Yusof

**Affiliations:** 1 Department of Mechanical Engineering, Faculty of Engineering, University of Malaya, 50603 Kuala Lumpur, Malaysia; 2 Department of Biomedical Engineering, Faculty of Engineering, University of Malaya, 50603 Kuala Lumpur, Malaysia; 3 Centre of Advanced Manufacturing and Material Processing, Lingkungan Budi, University of Malaya, 50603 Kuala Lumpur, Malaysia

**Keywords:** nanoparticle addition, composite solder, intermetallics compound, lead free solder joint

## Abstract

Nanocomposite lead-free solders are gaining prominence as replacements for conventional lead-free solders such as Sn–Ag–Cu solder in the electronic packaging industry. They are fabricated by adding nanoparticles such as metallic and ceramic particles into conventional lead-free solder. It is reported that the addition of such nanoparticles could strengthen the solder matrix, refine the intermetallic compounds (IMCs) formed and suppress the growth of IMCs when the joint is subjected to different thermal conditions such as thermal aging and thermal cycling. In this paper, we first review the fundamental studies on the formation and growth of IMCs in lead-free solder joints. Subsequently, we discuss the effect of the addition of nanoparticles on IMC formation and their growth under several thermal conditions. Finally, an outlook on the future growth of research in the fabrication of nanocomposite solder is provided.

## 1. Introduction

In an electronic packaging process, solder joints are employed as electrical and thermal connection as well as mechanical support for the soldering of electronic components to substrates. The reliability of solder joints is highly determined by the intermetallic compound (IMC) formed at the joint interface. A thin layer of an IMC may promote wettability between the solder and substrate, while an excessive formation of an IMC may weaken the joint as the IMC is brittle in nature [1]. A thick IMC layer will eventually lead to brittle fractures especially when subjected to different thermal conditions such as thermal aging and thermal cycling processes due to the thermal expansion mismatch between components and substrates [2]. Therefore, it is crucial to identify the formation of the IMC during soldering, and its growth behavior when the as-soldered joint is subjected to the above mentioned thermal conditions.

As global legislation has restricted the use of lead in electronic products due to health and environmental concerns, the use of lead-free solders has been proposed to replace lead-containing solders in the packaging process of electronic devices and components [3–5]. Among various solder alloy families, ternary Sn–Ag–Cu (SAC) lead-free solders are considered as one of the most promising substitutes as they possess a modest melting point, good wettability, fatigue resistance, and mechanical properties [6–8]. However, SAC solders possess a generally higher melting temperature and tin (Sn) content compared to lead-containing solders. Thus, the formation and growth of the IMC layer are more rapid in a SAC solder joint, resulting in brittle fractures and the reduced thermal fatigue life of the joint [9–13].

When SAC solder is used in microelectronic packaging which requires micron-scaled solder joints to be packaged in a narrow space, the joints are expected to be less reliable. These micron-scaled solder joints are exposed to a higher service temperature as high performance and multi-functional devices require high input and output connections, and thus lead to excessive growth of brittle IMC in the joint interface which may deteriorate joint reliability [14]. As the service temperature increases, the thermal expansion mismatch between the electronic components and substrate becomes larger. Solder joints have to sustain higher strains and thus, become more vulnerable when subjected to mechanical shock loading such as the dropping of devices [15]. Furthermore, the rapid on–off switching of devices will easily induce thermal cyclic fatigue failure in the micron-scaled joint [16]. Therefore, these factors have driven an urgent need to develop new or high performance SAC solders which can withstand high-temperature service conditions.

Recent studies have shown that one of the viable approaches is to develop a nanocomposite solder by adding a small amount of metallic or ceramic nanoparticles into the SAC solder [17–19]. These nanocomposite solders are found to have similar melting temperatures to SAC solders. Thus, they can replace SAC solders in industry without the need to purchase any new equipment or change the process parameters, which is very cost effective. The nanoparticles work by dispersing in the solder matrix and are distributed at the IMC grain boundary after soldering. Literature studies have shown that the dispersed nanoparticles can strengthen the solder matrix [20–22], refine the IMC formed in the joint interface, and suppress IMC growth while subjected to thermal cycling and thermal aging [23–25]. Accordingly, these findings strongly suggest the use of nanocomposite solders as a future solder material in industry.

In this review, we will discuss the current state of the effect of nanocomposite solders on IMC formation at the joint interface during soldering, and the IMC growth behavior under several thermal conditions. Before that, the fundamental studies of IMC formation between SAC solders and copper (Cu) substrates will be reviewed in order to explain the effect of the additional nanoparticles later on. A Cu substrate is chosen because it has been extensively used as a common conductor in the semiconductors or electronics industries due to its good solderability and excellent thermal conductivity [26, 27].

## 2. Fundamental studies of IMCs formation and growth in SAC/Cu solder joint interface

### 2.1. Introduction of soldering process

Soldering is a process in which two or more substrates are joined together by flowing a molten solder into the joint clearance. This process does not involve melting of the substrate as the solder used has a lower melting point (also referred as liquidus temperature) than the substrate. Hence, the reaction between solder and substrate during soldering is a liquid/solid reaction. Due to this reaction, an IMC layer will be formed at the joint interface during soldering and subsequent cooling [28].

**Figure 1.**
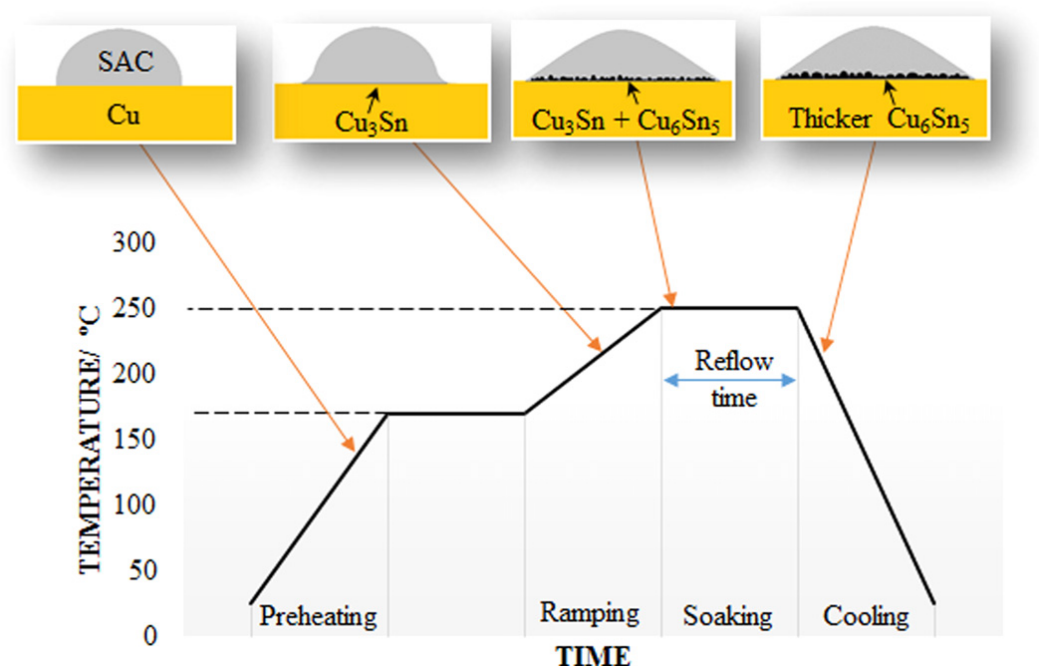
General soldering profile.

Basically, the general soldering regime for a SAC/Cu joint can be divided into four stages that are preheating, ramping, soaking, and cooling (figure 1). During preheating, the solder joint is heated to a temperature of 50 to 100 °C below the liquidus temperature of the SAC solder and is held at that temperature for a while. Next, the soldering temperature is ramped up to the peak temperature which is 30 to 50 °C above the solder liquidus temperature. The joint is then soaked in this temperature for a couple of minutes to reduce the temperature difference between the substrates and solder. Lastly, it is cooled down to room temperature and solidified to form a soldered joint. The preference peak temperature for SAC solder joining is usually around 250 °C as the liquidus temperature of SAC solders is approximately around 217 °C regardless of the solder composition. The formation of IMCs at each stage will be discussed in the following section.

### 2.2. Formation of IMCs during soldering at each stage (liquid–solid state diffusion)

#### 2.2.1. Preheating

During soldering, an interfacial reaction between the solder and substrate will only take place when the soldering temperature is slightly above the liquidus temperature of the solder. At this temperature, the solder is not fully liquefied, and thus the diffusion of Sn atoms from the solder to the contact region on the Cu substrate is slower. As the concentration of Cu atoms is locally higher than Sn atoms in the contact region, a very thin and fine *∊*-Cu_3_Sn (orthorhombic) IMC layer is formed at the interface [29].

#### 2.2.2. Ramping

**Figure 2.**
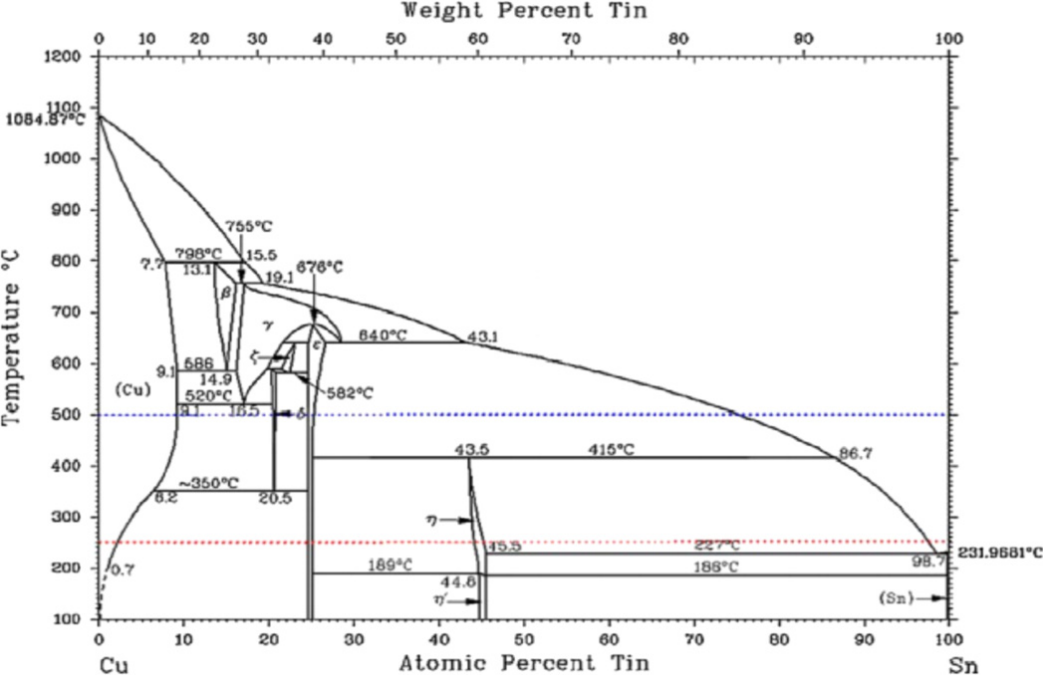
Binary Cu–Sn phase diagram. Adapted from Laurila *et al* [32], copyright 2010, with permission from Elsevier.

As the soldering temperature approaches 250 °C, the solder is fully molten and spread on the Cu substrate. The liquid–solid state diffusion between the elements of the molten solder and the solid substrate will take place at this time. More Sn atoms will be supplied to the contact region and form a coarse *η*-Cu_6_Sn_5_ (hexagonal) IMC layer on top of the fine *∊*-Cu_3_Sn IMC layer [12, 29–31]. The reported IMC formation mechanism is conformed to the binary Cu–Sn phase diagram shown in figure 2.

The morphology of *η*-Cu_6_Sn_5_ IMC grains strongly depends on the composition of the solder used. The grain is hemispherical or scallop-shaped when a near-eutectic or eutectic solder is used. On contrary, the grain is faceted when the solder composition is far away from the eutectic region [33, 34]. Moreover, the morphology of the *η*-Cu_6_Sn_5_ IMC grains may not be uniform along the SAC/Cu joint interface due to the uneven distribution of Cu atoms in the joint. The IMC layer had an elongated scallop shape in some studies due to excessive Cu atoms at certain regions in the joint [32, 35].

#### 2.2.3. Soaking

After reaching the peak temperature, the solder assembly will be held at this temperature for a short period time (which is called dwell or reflow time) in order to obtain a uniform temperature distribution throughout the assembly and to allow the molten solder to spread or wet the substrate more thoroughly. The *η*-Cu_6_Sn_5_ IMC grains will continue to grow during this period of time [18, 36]. Several IMC growth mechanisms at this stage have been reported and they will be discussed in the next section.

#### 2.2.4. Cooling

When the joint is subjected to subsequent cooling, the solubility of Cu atoms from the solid substrate to molten solder decreases as the temperature decreases. When the temperature drops below the liquidus temperature of SAC solder, the diffused Cu atoms are precipitated locally and nucleated heterogeneously on top of the existing *η*-Cu_6_Sn_5_ IMC interface due to the lower energy state requirement. Therefore, the *η*-Cu_6_Sn_5_ IMC grains become thicker and yet maintain the scallop morphology after the joint solidified completely [36]. On the other hand, the change in thickness of the *∊*-Cu_3_Sn IMC layer throughout the soldering has been reported to be insignificant as it is found to be left as a very thin IMC layer, and sometimes could not be observed by scanning electron microscopy due to a low soldering temperature or short soldering time [12, 30].

### 2.3. Growth mechanism of IMC during liquid–solid state diffusion

#### 2.3.1. *∊*-Cu_3_Sn IMC

During the soaking stage, the *∊*-Cu_3_Sn IMC and *η*-Cu_6_Sn_5_ IMC layers will continue to grow and have been reported to have different growth mechanisms at different reflow times. For the growth of the *∊*-Cu_3_Sn IMC, most of the works indicated that this IMC layer grew slightly with an increasing reflow time, forming a thin and planar morphology underneath the *η*-Cu_6_Sn_5_ IMC grains [18, 37, 38]. This is because a higher activation energy is required to form this IMC compared to the *η*-Cu_6_Sn_5_ IMC. The effect of activation energy on this IMC growth will be discussed in a later section.

#### 2.3.2. *η*-Cu_6_Sn_5_ IMC

For the growth mechanism of the *η*-Cu_6_Sn_5_ IMC layer, it was reported that their mechanism is varied with the reflow time [18, 36, 37, 39]. Therefore, the joints are subjected to a prolonged reflow time in order to identify the growth mechanism of the *η*-Cu_6_Sn_5_ IMC during the liquid–solid state diffusion.

**Figure 3.**
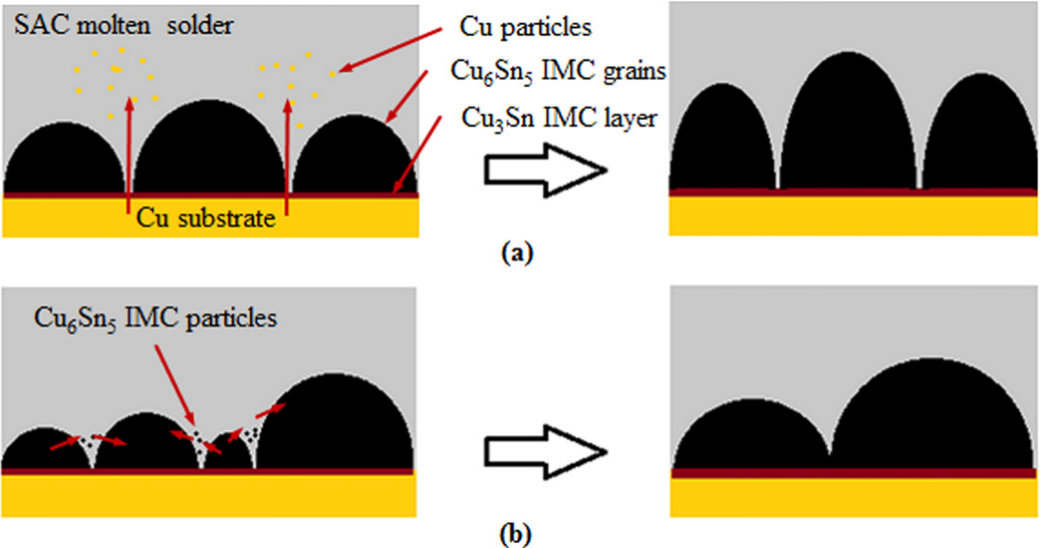
Schematics of (a) grain thickening and (b) grain coarsening.

From the work of Tang *et al* [18], Gagliano and Fine [37], Qu *et al* [40], and Zhang *et al* [39], it is noted that the *η*-Cu_6_Sn_5_ IMC growth during a short reflow time (less than 1000 s) is dominated by grain thickening and coarsening. At this time, the newly formed *η*-Cu_6_Sn_5_ IMC grains have a scallop shape and are separated from each other. This allows the Cu atoms from the substrate to diffuse through the channels between the separated grains, forming *η*-Cu_6_Sn_5_ IMC particles in the solder matrix. These particles are deposited on larger *η*-Cu_6_Sn_5_ IMC grains according to the Gibbs–Thomson effect, which states that the solubility of a particle is higher when the particle size or curvature is smaller [41, 42]. Therefore, the grains grow upward or thicken, as illustrated in figure 3(a). At the same, the grains become larger (grain coarsening) due to the Ostwald ripening effect, which describes the dissolution of smaller adjacent IMC grains into molten solder and then redeposition on the larger adjacent grains [43]. The mechanism is illustrated in figure 3(b). As the reflow time increased, the IMC grains continued to thicken and coarsen and finally closed off the channels between the grains.

**Figure 4.**
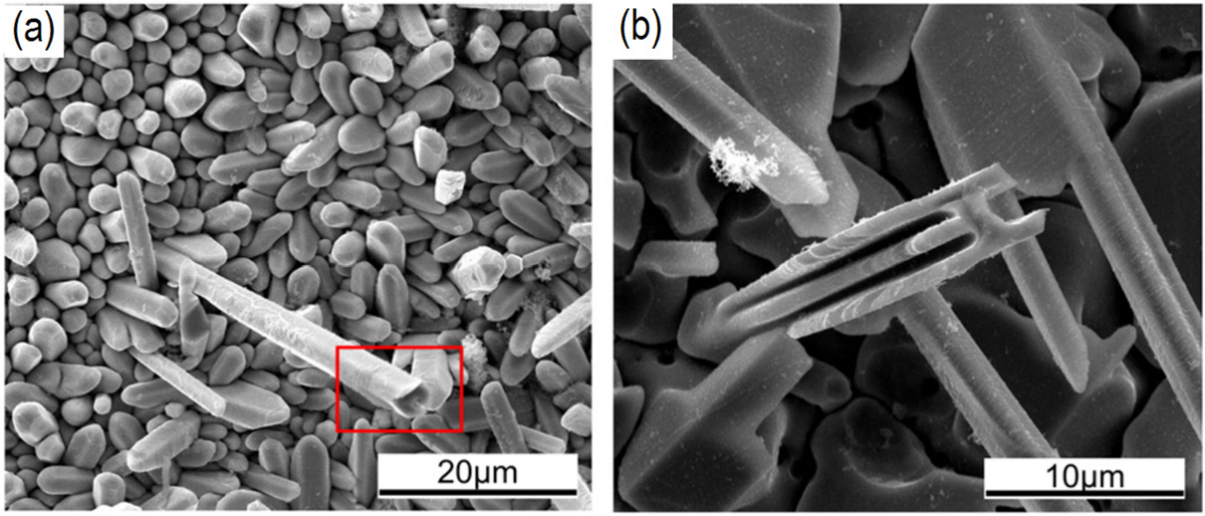
Morphology of Cu_6_Sn_5_ whiskers; (a) hexagonal; (b) acicular. Adapted from Tian *et al* [45], copyright 2014, with permission from Elsevier.

When the reflow time exceeded 1000 s, the channels between the IMC grains are completely pinched off, which forces the Cu atoms to diffuse through the thick IMC layers and therefore reduce their diffusion rate. Hence, the Cu_6_Sn_5_ IMC grains are more likely to coarsen rather than thicken. However, the coarsening rate of Cu_6_Sn_5_ grains is also reduced due to the formation of Cu_6_Sn_5_ IMC whiskers on top of the existing scallop grains. This is caused by a small amount of excessive Cu atoms which are supposedly present in the molten solder between scallops, and have flowed into the region beyond the grains by convection. They reacted with the Sn atoms and grew on the tops of the scallops to form cusps instead of larger grains. As the reflow time increased, grain coarsening is further hindered as these cusps elongated into hexagonal shaped whiskers that are hollow, with flat ends and sometimes in acicular shapes (refer to figure 4)[44, 45].

When the reflow time is further prolonged, the growth of the Cu_6_Sn_5_ IMC is mainly dominated by grain coarsening, according to Gagliano and Fine [37]. The diffusion of Cu atoms is hindered by the growth of the *∊*-Cu_3_Sn IMC and *η*-Cu_6_Sn_5_ IMC layers. The only source for grain coarsening mainly originates from the broken whiskers which are caused by the turbulence within the molten solder. The morphological change of both Cu_6_Sn_5_ and Cu_3_Sn IMCs at different reflow times has been summarized in table 1.

**Table 1.** IMC morphological change during soldering.

Type of IMC	Morphology of IMC at different reflow times		
	Short (<1000 s)	Long (>1000 s)	Prolonged
Cu_6_Sn_5_ (hexagonal)	Continuous scalloped (due to the grain thickening and coarsening mechanism)	Scalloped grains coarsen (dominant) and thicken, formation of Cu_6_Sn_5_ whiskers (either hollow hexagonal or acicular shape) on top of scalloped grains	Scalloped grains continue to coarsen, long Cu_6_Sn_5_ whiskers may break and flow into the solder matrix
Cu_3_Sn (orthorhombic)	Planar, very thin layer	Planar, thin layer	Planar, thin layer

### 2.4. Growth of IMCs during solid-state diffusion

During actual working conditions, the soldered joints are subjected to solid-state aging conditions such as the continuous use of electronic equipment, temperature storage, and power on–off cycles [46, 47]. The existing IMCs in the solid joint will continue to grow by solid-state diffusion under such thermal conditions. Therefore, thermal aging and thermal cycling tests are commonly used in research to simulate the alteration of joint microstructure and properties while under actual working conditions [47]. In this section, we will first discuss the growth behavior of IMCs present in SAC/Cu solder joints while subjected to thermal aging. The IMCs’ growth during thermal cycling will be discussed in the next section.

#### 2.4.1. Thermal aging

Thermal aging testing (also known as isothermal aging) is used to observe the degradation of joint properties when the soldered joint is subjected to a constant temperature for over a long period of time. During thermal aging, the as-soldered joint is thermally aged for up to 2000 h, within the aging temperature range of 100 to 170 °C [3, 17, 48, 49]. As mentioned earlier, the scallop-shaped *η*-Cu_6_Sn_5_ (hexagonal) and thin and fine *∊*-Cu_3_Sn (orthorhombic) IMC layers are the two main IMC layers present in the as-soldered joint. The thickness of both IMC layers increases during the thermal aging test due to solid-state diffusion between Cu and Sn atoms. The morphology of the scallop *η*-Cu_6_Sn_5_ IMC becomes planar as the channels between scallops provide convenient paths for the diffusion of Cu atoms. Thus, the IMC grows faster at the channels between scallops rather than on the top surface of the scallops during the initial aging stage [26, 50]. In other words, the Cu_6_Sn_5_ IMC layer becomes planar rather than thickening in such conditions. On the contrary, the Cu_3_Sn IMC layer remains planar but thickens significantly with increasing aging temperature and time [50, 51]. However, there might be a change in the Cu_3_Sn lattice structure, as Mookam and Kanlayasiri [47] reported that hexagonal Cu_3_Sn was also present in the joint interface after aging for 1000 h at 100 °C. The alteration of the lattice structure may affect the slip mechanism of the joint when subjected to mechanical forces.

Additionally, the formation of Kirkendall voids at the Cu/Cu_3_Sn interface and in the Cu_3_Sn IMC layer has also been observed in thermally aged joints. This is due to the faster diffusion of Cu than Sn during thermal aging conditions, in which atomic level vacancies left by the migrating Cu atoms on the substrate are not filled by Sn atoms. These vacancies coalesce into voids which are named Kirkendall voids [51, 52]. The density of the voided area increases with aging time and temperature which causes brittle fractures in the joint [49, 52–54].

The growth of the Cu_3_Sn IMC layer during thermal aging is mainly governed by the inter-diffusion between Sn atoms diffused through the Cu_6_Sn_5_ IMC layer and Cu atoms from the metal substrate at the Cu/Cu_3_Sn interface [47, 51, 55]. The interfacial reaction can be expressed by equation (1) [47]. The growth of the Cu_3_Sn IMC layer may also be contributed to by Cu atoms diffused through the Cu_3_Sn to Cu_3_Sn/Cu_6_Sn_5_ interface [26, 47, 51, 53]. The reaction is described in equation (2).

For the Cu_6_Sn_5_ IMC, its growth is governed by the reaction (as stated in equation (3)) between Cu and Sn atoms available in the Cu_6_Sn_5_/solder interface. Due to the reaction in equation (2), the amount of Cu atoms diffused from the metal substrate to the Cu_6_Sn_5_/solder interface is greatly reduced as the Cu_3_Sn layer thickens with aging time. At the same time, the Cu supply from the solder is limited as most of the Cu atoms have been used to form Cu_6_Sn_5_ IMC particles in the solder matrix [26]. Therefore, the growth of the Cu_3_Sn IMC is more significant compared to that of the Cu_6_Sn_5_ IMC during thermal aging

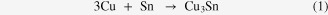


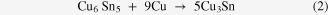


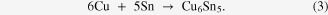


However, when the supply of both Cu and Sn atoms is sufficient, the growth of IMCs in the solder joint is dependent on the activation energy required to form the particular IMC. Based on literature [26, 47, 56, 57], one of the famous approaches is to calculate the activation energy of the Cu_6_Sn_5_ and Cu_3_Sn IMCs that is derived from the data acquired from the thermal aging test. The details of the derivation are presented in the following section.

##### 2.4.1.1. Calculation of IMCs’ activation energy

The activation energy of a reaction is the minimal energy required for the reaction to occur. The lower the activation energy, the higher the thermodynamic stability of the reaction. In other words, the growth of an IMC is faster when the activation energy required is lower, but with the condition that there is a sufficient supply of reactants. Over the years, studies have been conducted to determine the activation energy for the formation of Cu_6_Sn_5_ and Cu_3_Sn IMCs and the following is the summary of the calculation method.

As the growth of IMCs during thermal aging is governed by the solid-state diffusion mechanism, or more precisely the lattice diffusion dominated diffusion mechanism [50], the relationship between the IMC layer thickness and aging time can be expressed by the following equation [26, 27].

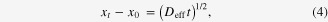
where the *t* is aging time, *x*_0_ is the IMC thickness of the as-soldered joint, *x*_*t*_ is the IMC thickness at aging time *t*, and *D*_eff_ is the effective diffusion coefficient of the IMC during thermal aging. By plotting the graph of IMC thickness, *x*_*t*_ against the square root of the aging time, *t*^1/2^, the *D*_eff_ for the both IMCs can be determined from the slope of the linear curve. The activation energies for each IMC can be determined by the Arrhenius equation as expressed in equation (5)

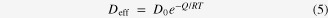
where *D*_0_ is the temperature-independent diffusion coefficient, *Q* is the activation energy, *R* is the universal gas constant, and *T* is the absolute temperature in Kelvin (K). By taking the logarithm of equation (5), the equation can be expressed as follows:

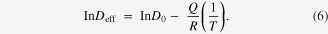


The activation energy of the IMC can be obtained by calculating the slope of the plot of the efficient diffusion coefficient (In*D*_eff_) against the inverse of the aging temperature (1/*T*). The activation energy of Cu_6_Sn_5_, Cu_3_Sn and (Cu_6_Sn_5_ + Cu_3_Sn) IMCs obtained from several studies are listed in table 2. From the table, the activation energy of the Cu_6_Sn_5_ IMC phase is generally the lowest while the Cu_3_Sn IMC is the highest regardless of the solder composition. This indicates that the formation of the Cu_6_Sn_5_ IMC is more thermodynamically stable than the Cu_3_Sn IMC. These data are consistent with the observation in which the Cu_6_Sn_5_ IMC layer thickness is always thicker than the Cu_3_Sn IMC thickness. The variation of the activation energy might be attributed to the use of different types of Cu which have different grain sizes, energy states and impurities that can influence the diffusivity of Cu atoms [51, 58].

**Table 2.** Activation energies obtained from literature studies.

			Activation energy, kJ mol^−1^			
Solder	Aging temperature, °C	Aging time, h	Cu_6_Sn_5_	Cu_3_Sn	(Cu_6_Sn_5_ + Cu_3_Sn)	Ref.
SAC 037	100–170	0–1000	51.59	119.04	80.65	[47]
SAC 305	100–170	0–672	58.3	114.7	75.1	[56]
SAC 355	120–180	0–2000	69.42	91.88	79.79	[57]
SAC 387	100–140	0–720	61.4	105.8	73.5	[26]
SAC 387	100–150	0–1000	—	119.8	—	[51]
SAC 387	75–175	0–1008	52.27	80.69	77.7	[17]

#### 2.4.2. Thermal cycling (solid-state diffusion)

In actual working or service conditions, electronic products are usually subjected to cyclic temperature changes rather than constant temperatures. The coefficient of thermal expansion (CTE) mismatch between the electronic components and circuit board will generate cyclic thermal stresses and strains in the SAC/Cu solder joint leading to thermal fatigue failures [26, 50]. This gives rise to the importance of identifying the growth behavior of the IMCs under thermal cycling conditions in order to improve the joint reliability under actual working conditions.

The thermal cycling test (also called the thermo-mechanical fatigue test) is used to determine the joint’s ability to resist extremely high and low temperatures, as well as to withstand cyclical exposures to these temperature extremes. It is used to accelerate the occurrence of joint fatigue failures under cyclic stress and strain [26, 59]. When the joint is subjected to thermal cyclic stresses, thermal activated diffusion will take place in the solder joint interface. According to Teo and Sun [59], the IMC layers at the Cu/SAC interface will grow in order to relieve the induced residual stress, and the growth corresponds to the magnitude of the induced stress.

During a thermal cycling test, the as-soldered joint is heated in a furnace with a temperature range of −40 to +150 °C for 0 to 2000 cycles in general. The ramp rate is around 10 °C min^−1^ and the dwell time at each extreme temperature is around 10 min. The joint will be air cooled to room temperature after reaching the desired thermal cycles [60]. Similar to the thermal aging case, the thickness of both *η*-Cu_6_Sn_5_ and *∊*-Cu_3_Sn IMC layers increases with increasing numbers of thermal cycles, and the morphology of *η*-Cu_6_Sn_5_ IMC changes from scallop-like to planar [26, 61]. However, Shen *et al* [50] reported that the growth of both IMCs is faster during thermal cycling if compared to that during thermal aging. Moreover, the *η*-Cu_6_Sn_5_ IMC grew faster than *∊*-Cu_3_Sn IMC during thermal cycling which is totally opposite to that during thermal aging. The authors compared the growth behavior during thermal aging and thermal cycling by using equivalent aging duration parameters, in which *t*_eff_ is defined as the total accumulated dwell duration. The growth of total IMC layers under both thermal conditions can be explained by the following equation:

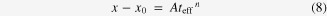
where *x* is the total thickness of both IMC layers at time *t*_eff_, *x*_0_ is the as-soldered total thickness of the IMC layer, *A* is the growth constant, and *n* is the time exponent. The *n* values obtained for thermal aging and thermal cycling tests are 0.51 and 0.6 respectively, which indicate that IMC growth is faster during thermal cycling. The increase in IMC growth rate is contributed by (1) lattice diffusion and grain boundary diffusion paths for Sn atoms due to the dynamic recrystallization occurring during thermal cycling, and (2) faster diffusion of Cu atoms due to higher thermal mechanical stress.

When the as-soldered joint is subjected to thermal cycling, the CTE mismatch between the solder and substrate induces thermal stress and strain in every cycle. Dynamic recrystallization occurs and new grains form in the joint to release the stress. When the thermal cycle is repeated, new deformation will occur before the existing grain grows and new grains will be formed. Therefore, the number of grains and grain boundaries increases but the grain size decreases with increasing thermal cycles. As a result, the Sn atoms in the solder can diffuse into the solder/IMC interface by lattice diffusion and grain boundary diffusion during thermal cycling. There is an additional diffusion path during thermal cycling compared to thermal aging which is dominated by lattice diffusion only [50]. Moreover, grain boundary diffusion is claimed to be faster than lattice diffusion. On the other hand, the higher thermal mechanical stress generated during thermal cycling resulted in faster diffusion of Cu atoms into the joint [62]. When temperature variation increased, thermal mechanical stress increased, and thus the diffusivity of Cu atoms increased during each cycle. Therefore, both IMCs grew faster during thermal cycling and the growth of *η*-Cu_6_Sn_5_ is faster than the Cu_3_Sn IMC due to the sufficient supply of Cu and Sn atoms as well as the lower activation energy [50]. This is consistent with the observation of Zhang *et al* [26] and Han *et al* [24]. As a summary, the growth of IMCs in the as-soldered, thermally aged and thermally cycled solder joint is compared and summarized in table 3.

**Table 3.** Comparison of IMC growth at different thermal conditions.

	Growth of IMC at different thermal conditions		
Type of IMC	As-soldered	Thermally aged	Thermally cycled
Cu_6_Sn_5_	Scalloped morphology, IMC grown faster than Cu_3_Sn	Planar morphology, IMC layer grown thicker	Planar morphology, IMC grown faster than Cu_3_Sn. Growth rate is faster than during thermal aging
Cu_3_Sn	Planar morphology, insignificant IMC growth	Planar morphology, IMC grown more significantly than Cu_6_Sn_5_, Kirkendall voids may form at Cu_3_Sn/Cu interface or within the IMC layer	Planar morphology, IMC layer grown thicker. Growth rate is faster than during thermal aging

## 3. Effect of nanoparticle addition on SAC–X/Cu joint interface

The purpose of adding nanoparticles into lead-free solders is to strengthen the solder by particle dispersion which can improve the solder deformation resistance by impeding the movements of dislocation and pin grain boundaries in the solder matrix [63, 64]. Besides, the presence of nanoparticles can prevent excessive IMC growth by restricting the diffusion activity of the relevant elements in the joint [17, 32, 35, 65]. A number of nanoparticles have been investigated and they can be categorized into metallic, ceramic, and carbon nanotubes. However, the literature about their effects on the IMCs growth in the joint interface is still very limited. Thus, the effect of the addition of selected nanoparticles in the SAC–X/Cu joint will be systematically reviewed in detail in this section. In general, nanocomposite solders are prepared by manually mixing the nanoparticles (in powder form) with the SAC solder paste under atmospheric condition, followed by the blending of the nanocomposite solder paste to ensure the nanoparticles are uniformly dispersed in the solder paste. It is noted that the added nanoparticles may not be completely incorporated in the SAC solder after soldering. The particles may be rejected, engulfed or entrapped in the molten solder, depending on the interaction mechanisms between the particles and solder [17, 66]. The particles may sometimes be dispersed into the solder flux rather than the solder matrix. Therefore, for the ease of comparison, we will only consider the nominal percentage of the addition in the solder paste instead of the actual incorporated amount.

### 3.1. Metallic nanoparticles

#### 3.1.1. Nickel (Ni)

Several studies have reported that the addition of Ni particles into lead-free solders could enhance the mechanical properties of the solder. Niranjani *et al* [67] observed that the hardness and creep resistance of the composite solder have been improved with the addition amount of 0.5 wt% Ni nanoparticles. Yao *et al* [68] and Tay *et al* [17] reported that Ni-containing composite solders possessed better wettability. The effects of Ni particles on the IMC growth in the solder joint during soldering and under different thermal conditions have also been reported and will be discussed next [17, 23, 53, 69, 70].

During soldering, Ni particles altered the composition of Cu_6_Sn_5_ IMC in the joint by substituting the Cu atoms in the IMC grains and forming a (CuNi)_6_Sn_5_ IMC. Yoon *et al* [71] and Laurila *et al* [72] found that the atomic size of Ni (0.125 nm) is very similar to Cu (0.128 nm) and both have a face-centered cubic (fcc) lattice structure. Therefore, Ni atoms can substitute the Cu atoms in the IMC lattice without distorting the lattice structure. Besides, the energy required to form the (CuNi)_6_Sn_5_ IMC is lower than the Cu_6_Sn_5_ IMC which indicates that the (CuNi)_6_Sn_5_ IMC possesses higher stability.

The morphology of (CuNi)_6_Sn_5_ IMC grains is also similar to Cu_6_Sn_5_ IMC grains but are more refined and the IMC layer is thinner compared to Cu_6_Sn_5_ IMC [23]. According to Liu *et al* [53], the grain refinement is greater when the amount of addition is increased from 0.5 wt% to 2 wt%. The grain refinement is contributed by the stronger affinity between Ni and Sn (the affinity between Cu and Sn is weaker) and lower energy for the formation of the (CuNi)_6_Sn_5_ IMC. The activation energy of the (CuNi)_6_Sn_5_ IMC is reported to be 49.3 kJ mol^−1^ [69] which is lower than that of the Cu_6_Sn_5_ IMC (refer to table 2). Therefore, these resulted in the higher nucleation rate of the IMC and thus further refined the IMC grains.

**Figure 5.**
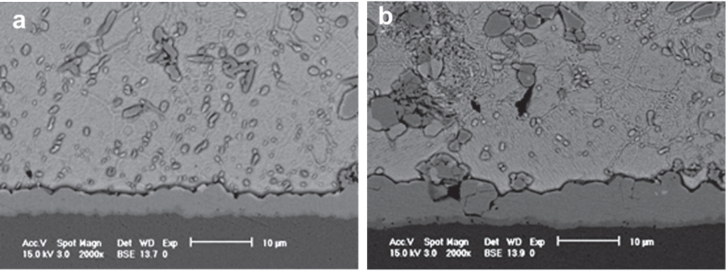
Cross-sectional scanning electron microscopy (SEM) micrographs of SAC–0.5Ni/OSP–Cu joint interface for aging times at 150 °C of (a) 10 days and (b) 40 days. Adapted from Gain and Chan [69], copyright 2012, with permission from Elsevier.

When the joint is subjected to prolonged reflow and thermal aging, the (CuNi)_6_Sn_5_ IMC layer becomes thicker and more planar, which is similar to the growth of the Cu_6_Sn_5_ IMC in a SAC/Cu joint under the same conditions [17, 53, 69]. The only difference is the growth of the Cu_3_Sn IMC layer, which is much slower during thermal aging compared to those in a SAC/Cu joint. This is because the (CuNi)_6_Sn_5_ IMC is more thermodynamically stable than the Cu_6_Sn_5_ IMC and thus it will not easily decompose to form the Cu_3_Sn IMC as stated in equation (3) [56, 73]. The microstructures of thermally aged solder joints are shown in figure 5.

**Figure 6.**
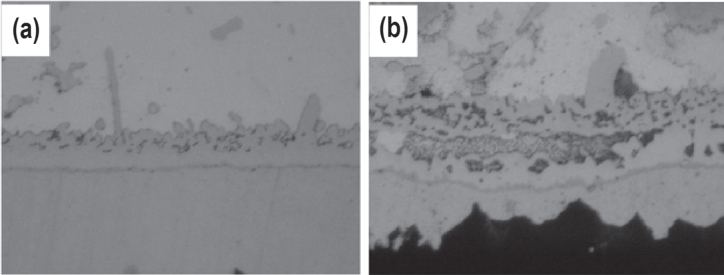
Cross sectional SEM micrographs of SAC–2.0Ni/Cu interfaces aged at 150°C for (a) 200 h and (b) 1000 h. Adapted from Liu *et al* [53], copyright 2009, with permission from Elsevier.

When the joint with a Ni particle addition of 2.0 wt% is subjected to an aging time of 1000 h, two distinct (CuNi)_6_Sn_5_ IMC regions were found in the joint in which a small amount of solder is entrapped within the layer (refer to figure 6(b)) [53]. The dense region which has a lower Ni content is close to the Cu substrate while the loose region which has a higher Ni content is close to the solder. It was reported that the dense region is formed during soldering, while the loose region is formed during thermal aging as its thickness increased with increasing aging time. The formation of the loose IMC region is more likely due to the precipitation of increasing amounts of the (CuNi)_6_Sn_5_ IMC particles in the solder matrix as more Ni particles were present in the matrix in this case. Unfortunately, there is no report on the effect of such structures on the solder joint’s properties.

In summary, the addition of Ni particles can significantly suppress the growth of the Cu_3_Sn IMC layer during thermal aging and prolonged soldering. However, the growth of the (CuNi)_6_Sn_5_ IMC layer can be stimulated by these Ni particles in all soldering and aging conditions as the (CuNi)_6_Sn_5_ IMC possesses a lower activation energy than the Cu_6_Sn_5_ IMC. On the solder itself, excessive Ni addition will increase the viscosity of the composite solder, which inhibits its spreading on a substrate [17, 68].

#### 3.1.2. Aluminum (Al)

According to Shnawah *et al* [74], aluminium (Al) has been proposed as an addition element in SAC solder due to its low cost and non-hazardousness towards the environment. Li *et al* [12] revealed that the IMC growth can be significantly reduced with 1 wt% of Al element alloyed in the SAC solder by reducing the activity of Sn and Cu during soldering and thermal aging. The *η*-Cu_6_Sn_5_ IMC formed was thinner and a layer of *η*_2_-AlCu IMC was formed in the solder bulk and then migrated towards the solder joint interface. This IMC layer could replace the existing *η*-Cu_6_Sn_5_ and *∊*-Cu_3_Sn IMCs, and be transformed to a *δ*-Al_2_Cu_3_ IMC when reacted with Cu. It was finally dispersed into the solder bulk, and thus reduced the growth of *η*-Cu_6_Sn_5_ and *∊*-Cu_3_Sn IMCs. The formation of Al–Cu particles increased with increased Al element content [75].

When Al is added into solder as nanoparticles instead of alloying element, these nanoparticles do not dissolve or interact with the Cu_6_Sn_5_ IMC to form an AlCu IMC layer [23, 69]. Instead, Gain and Chan [69] found that fine spherically-shaped Sn–Ag–Al IMC particles are formed in the *β*-Sn solder matrix when added at 0.5 wt% Al nanoparticles in a SAC/Cu solder joint. In another research of Gain *et al* [76], the same IMC particles are observed to be distributed on top of the (Cu,Ni)_6_Sn_5_ IMC layer while reflowing SAC–*x*Al (*x* = 0.5 to 3.0 wt%) solder on a Au and Ni metallized Cu pad. The authors also suggest that these IMC particles enhanced the strength and hardness of the solder. Furthermore, the fracture mode of SAC/Au–Ni metallized Cu joint has been changed from the brittle to ductile mode due to the formation of Sn–Ag–Al IMC particles on top of the (Cu,Ni)_6_Sn_5_ IMC layer.

**Figure 7.**
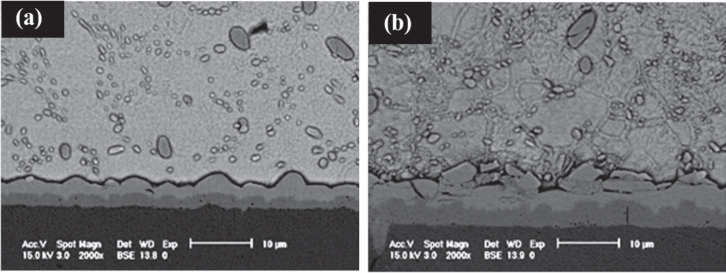
Cross sectional SEM micrographs of a SAC–0.5Al/OSP–Cu joint interface for aging times of (a) 10 days and (b) 40 days at 150 °C. Adapted from Gain and Chan [69], copyright 2012, with permission from Elsevier.

When the Al-containing composite solder joint is subjected to prolonged reflow and thermal aging, both Cu_6_Sn_5_ and Cu_3_Sn IMC thicknesses increase but the growth of the Cu_3_Sn IMC is slower. The calculated activation energy for the total (Cu_6_Sn_5_ + Cu_3_Sn) IMC in the joint is 55.1 kJ mol^−1^ which is much lower if compared to the data presented in table 2. In other words, Al nanoparticles can effectively suppress the IMC growth in the composite solder joint. The joint microstructure after thermal aging is shown in figure 7. By comparing the thickness of IMC layers in figure 7 with those in figure 5, the effectiveness of Al nanoparticles in suppressing the IMC growth is apparently lower than that of Ni nanoparticles. This is because Al does not dissolve in the Cu_6_Sn_5_ IMC and thus, the IMC layer does not inhibit the diffusion of Cu atoms as effectively as (Cu,Ni)_6_Sn_5_ IMC does [69].

#### 3.1.3. Cobalt (Co)

Over the years, cobalt (Co) has been added to lead-free solders in the form of an alloying element and particle reinforcement. The Co-containing lead-free solders are found to have better shear ductility [77], strength [63], and thermal fatigue and creep resistance [78]. This may be attributed to the formation of a (Cu,Co)_3_Sn_2_ IMC in the solder matrix, which strengthens the solder by its dispersion effect.

When Co nanoparticles are added to a SAC/Cu solder joint, Co atoms substituted the Cu sites in the Cu_6_Sn_5_ IMC lattice structure, forming a new IMC layer, (Cu,Co)_6_Sn_5_ [48]. The situation is similar to the case of Ni addition because both elements have the same atomic radius. However, the lattice structure of Co is a hexagonal close-packed (hcp) structure, which is different from Ni and Cu (both in a fcc lattice structure). Hence, the thermodynamic affinity between Sn and Co is weaker than that between Sn and Ni but higher than that between Sn and Cu [73, 79]. In other words, the (Cu,Co)_6_Sn_5_ IMC is more thermodynamically stable than Cu_6_Sn_5_ IMC but less stable than (Cu,Ni)_6_Sn_5_ IMC.

**Figure 8.**
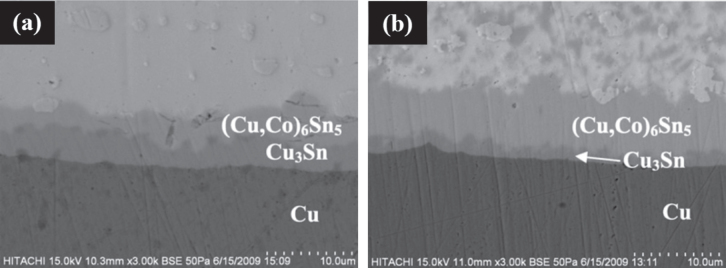
Cross-sectional SEM micrographs of the SAC–*x*Co/Cu joint interface after aging at 150 °C for 1008 h (42 days); (a) *x* = 0.5 wt%, (b) *x* = 1.5 wt%. Adapted from Haseeb and Tay [48], copyright 2011, with permission from Elsevier.

From the as-soldered joint, the (Cu,Co)_6_Sn_5_ IMC grains are more refined if compared to the Cu_6_Sn_5_ IMC grains in the SAC/Cu joint [23]. Therefore, the IMC layer looks more planar than scallop-like (refer to figure 8) [23, 48].

When the joint is subjected to thermal aging, a Cu_3_Sn IMC layer is observed and both (Cu,Co)_6_Sn_5_ IMC and Cu_3_Sn IMC layers grow thicker with increasing aging time. However, when the amount of Co addition increases, the growth of (Cu,Co)_6_Sn_5_ IMC is promoted while the growth of Cu_3_Sn IMC is suppressed (refer to figure 8). Haseeb and Leng [48] suggested that the suppression of the Cu_3_Sn IMC layer for Co addition is similar to that for Ni addition. Due to the stronger thermodynamic affinity between Sn and Co than Sn and Cu, the driving force for formation of the Cu_3_Sn IMC is reduced while the formation of the (Cu,Co)_6_Sn_5_ IMC is increased.

In summary, the influence of Co nanoparticles in SAC/Cu solder joint is very similar to that of Ni nanoparticles but the effectiveness of Co in suppressing the Cu_3_Sn IMC is slightly weaker due to weaker thermodynamic affinity. On the other hand, the effects of Co as nanoparticles addition on the IMC growth closely resembles those of Co [49, 77, 80]. From the research of Haseeb and Leng [48] and Amagai [23], we can conclude that even a very small addition of Co nanoparticles (as little as 0.03 wt%) is sufficient to induce beneficial changes in the growth of an IMC at the joint interface. Further research is required to conclude the optimum range of Co nanoparticle addition, as excessive addition of Co may cause excessive growth of the IMC and degrade the joint reliability [32]. This is evidenced by the work of Lee *et al* [63] where excessive growth of the (Cu,Co)_3_Sn_2_ IMC is observed when the addition of Co particles is up to 2.0 wt% which caused brittle fractures at the interfacial IMC in the joint.

#### 3.1.4. Molybdenum (Mo)

From the studies of Kumar *et al* [81], Mohankumar and Tay [64], and Rao *et al* [82], a Mo nanoparticle addition of up to 2.0 wt% in SAC solder is capable of improving the solder hardness and strength due to the formation of small and regularly shaped Mo–Sn IMC particles. These IMC particles dispersed in the *β*-Sn solder matrix which can prevent grain boundary sliding and restrict the dislocation movement of the bulk solder [67].

To date, the study of the influence of Mo nanoparticles on IMC growth in SAC/Cu joint interfaces is very limited. In fact, the paper of Haseeb *et al* [7] and Arafat *et al* [83] are the only sources of information. Haseeb *et al* [7] revealed that Mo nanoparticles do not interact with the existing Cu_6_Sn_5_ IMC to form a new IMC layer during soldering. However, these particles could retard and refine the growth of the Cu_6_Sn_5_ IMC during soldering. The morphology of the Cu_6_Sn_5_ IMC in the SAC–Mo/Cu joint interface is more refined and the IMC layer thickness is only half of those formed in SAC/Cu joint interfaces. The refinement of the IMC increased while the layer thickness decreased with increasing amounts of addition. The authors found that Mo nanoparticles tend to accumulate on the surface of Cu_6_Sn_5_ IMC scalloped grains and the channel between them rather than disperse in the solder matrix. This phenomenon may be explained by the density difference between the molten solder (6.99 g cm^−3^) and Mo nanoparticles (10.28 g cm^−3^) during soldering. The adsorbed Mo nanoparticles on the IMC grain surface reduced the ripening effect and thus inhibit the neighboring IMC grains from coalescing into larger grains. The presence of Mo nanoparticles in the channels reduced the diffusion and dissolution of Cu atoms from the metal substrate into the interface, and Sn atoms from the solder into the interface. Therefore, the Cu_6_Sn_5_ IMC layer in the SAC–Mo/Cu joint is thinner and smaller in grain size than that of the SAC/Cu solder joint. A similar observation was obtained by Arafat *et al* [83] who added different amounts of Mo nanoparticles (up to 5 wt%).

**Figure 9.**
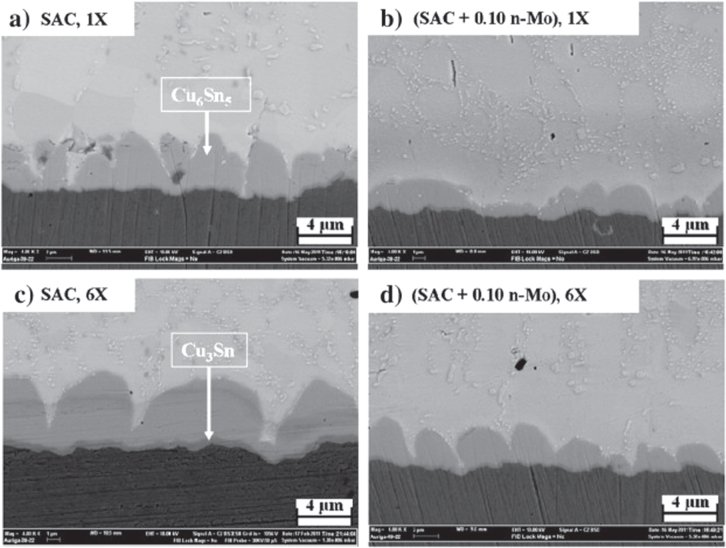
Cross sectional view of joint interface (a) as-soldered SAC/Cu, (b) SAC/Cu after 6 thermal cycles, (c) as-soldered SAC–0.1Mo/Cu and (d) SAC–0.1Mo/Cu after six thermal cycles. Adapted from Haseeb *et al* [7], copyright 2012, with permission from Elsevier.

As reported by Haseeb *et al* [7] and Arafat *et al* [83], both the Cu_6_Sn_5_ IMC and Cu_3_Sn IMC were present in the joint interface during thermal cycling and prolonged reflow time. Both IMCs grew thicker but the Cu_6_Sn_5_ IMC layer thickness was half of that obtained from the SAC/Cu joint, while the Cu_3_Sn IMC was just slightly thinner (refer to figure 9). Therefore, it can be said that Mo nanoparticles can effectively retard the growth of both Cu_6_Sn_5_ and Cu_3_Sn IMCs but its effectiveness in retarding the Cu_3_Sn IMC is much weaker compared to Ni and Co.

#### 3.1.5. Zinc (Zn)

Studies have reported that the addition of Zn as an alloying element and nanoparticle reinforcement have brought benefits towards the solder strength but deteriorated the solder wettability. The addition of Zn as an alloying element in SAC solder caused the formation of near spherical Cu_5_Sn_8_, Ag_5_Zn_8_, and AgZn_3_ IMC particles in the solder matrix [84]. Besides, it was reported that the grain size of primary *β*-Sn dendrites, and Ag_3_Sn and Cu_6_Sn_5_ IMC particles in the solder matrix was refined when SAC alloyed with Zn. The formation of Cu_5_Sn_8_ IMC particles can effectively retard the dislocation occuring in the solder and consequently improve the creep resistance. El-Daly and El-Taher [85] reported that the superior creep resistance is achieved with a 0.5 wt% Zn addition. The authors also reported that the addition of Zn has effectively reduced the undercooling of the solder, which may lead to a non-homogeneous distribution of the unwanted phases in the solder joint. The tensile strength of the Zn-containing solder is found to be higher than plain SAC solder if the addition of Zn is less than 2 wt%. However, the ductility of Zn-containing solder is decreasing with increasing Zn content due to the present of Ag_5_Zn_8_ IMC particles. Song *et al* [84] observed that the fracture of the Zn-containing solder is originated at the Ag_5_Zn_8_ IMC particles. A similar observation is reported by Lin and Chuang [86] who investigated the addition of 0.2 and 0.5 wt% Zn into SAC–Ce solder alloy. Formation of fine (Ce,Zn)Sn_3_ IMC particles in the bulk solder has improved the tensile strength but sacrificed the ductility of the Zn-containing solder. On the other hand, when Zn is added to SAC solder as nanoparticle reinforcement, it is noticed that the wettability of the composite solder is decreased [30, 87]. The deterioration of solder wettability may be caused by the oxidation of Zn nanoparticles during soldering, which increases the surface tension of the solder [88, 89]. The addition of nanoparticles may also increase the viscosity of the molten solder and therefore inhibit the spread of the molten solder on the substrate [14].

When Zn nanoparticles are added into a SAC/Cu solder joint, the amount of addition seems to be a very critical factor in controlling the IMC growth. Amagai [23] and Yahya *et al* [90] reported that by adding 0.05 and 0.1 wt% of Zn nanoparticles in the solder joint did not introduce any effect on the IMC layer thickness and grain size even after thermal cycling and thermal aging. When the addition amount increased to 1 wt%, a Cu_6_Sn_5_ IMC layer is observed but its thickness is thinner than that of the Zn-free solder joint [87]. When the Zn addition is further increased to 2 wt%, a new IMC layer, Cu_5_Zn_8_, is formed on top of the Cu_6_Sn_5_ IMC layer and in the solder matrix. Both Cu_6_Sn_5_ and Cu_5_Zn_8_ IMC layers grow slightly and the Cu_3_Sn IMC is not detected even when the joints are subjected to six reflow cycles. Furthermore, the growth of the Cu_6_Sn_5_ IMC layer is found to be decreased with increasing Zn content. Chan *et al* [87] attributed the suppression of Cu_6_Sn_5_ and Cu_3_Sn IMCs to the formation of the Cu_5_Zn_8_ IMC layer, which retards the diffusion of Cu to form Cu–Sn IMCs.

A similar observation was obtained by Kotadia *et al* [30] who alloyed 0.1 to 1.5 wt% Zn element into a SAC/Cu solder joint. Formation of a Cu_5_Zn_8_ IMC layer is observed on top of the Cu_6_Sn_5_ IMC layer when the Zn content is 1 wt%. However, this IMC layer broke off and massively moved into the solder matrix. The massive spalling of this IMC layer only occurs when the solder volume is limited, and such phenomena did not occur when soldering is performed in a solder bath. The authors noticed that the growth of Cu_6_Sn_5_ and Cu_3_Sn IMCs is suppressed significantly after thermal aging while the Cu_5_Zn_8_ IMC layer seems to remain almost the same.

As a summary, addition of Zn either as an alloying element or as nanoparticles addition can effectively retard the growth of Cu_6_Sn_5_ and Cu_3_Sn IMC layers by forming a Cu_5_Zn_8_ IMC layer to reduce the diffusion reaction between Sn and Cu atoms in the solder joint. The minimum effective addition of Zn element has to be approximately 1.0 wt% or else there might not be any effect on IMC growth in the joint.

### 3.2. Ceramic nanoparticles

#### 3.2.1. Alumina (Al_2_O_3_)

Composite lead-free solders with added Al_2_O_3_ nanoparticles have been reported to possess better microhardness, wettability [91], tensile strength [92, 93] creep resistance [20], and lower CTE [94]. The improvement in these properties is attributed to the dispersion of the nano-Ag_3_Sn IMC in the solder matrix. The grain size of the Ag_3_Sn IMC is refined in the composite solder matrix due to the adsorption of nano-Al_2_O_3_ particles on the Ag_3_Sn IMC grain surface during solidification of the solder. According to the theory of adsorption of surface active materials [22], the increase in adsorption of Al_2_O_3_ nanoparticles decreases the surface free energy of the Ag_3_Sn IMC grains and thus impedes the growth of these IMC grains. The morphology of the Ag_3_Sn IMC changed from needle-like to particle-like in the composite solder matrix [35, 94, 95]. However, Tsao *et al* [91] and Chuang *et al* [94] observed that the beneficial influences of nano-Al_2_O_3_ reinforcement are reduced when the amount of Al_2_O_3_ nanoparticles reaches 1.0 wt%.

Chang *et al* [95] investigated IMC growth in the SAC-0.5Al_2_O_3_/Cu joint interface under prolonged soldering and high temperature soldering. Al_2_O_3_ nanoparticles did not react with Cu or Sn atoms to form a new IMC at the interface but altered the morphology of the Cu_6_Sn_5_ IMC layer. A continuous scallop-shaped Cu_6_Sn_5_ IMC layer was observed to change to a discontinuous scallop-shape and become thinner after Al_2_O_3_ nanoparticles were added in the SAC/Cu joint. The formation of Cu_6_Sn_5_ IMC whiskers which occurred in high temperature soldered SAC/Cu joints was not found in the SAC–0.5Al_2_O_3_/Cu joint. The authors also reported that the activation energy of the IMC layer in the SAC–0.5Al_2_O_3_/Cu system was higher than that of the SAC/Cu system. The findings indicated that the growth of the Cu_6_Sn_5_ IMC layer was retarded by the addition of Al_2_O_3_ nanoparticles.

**Figure 10.**
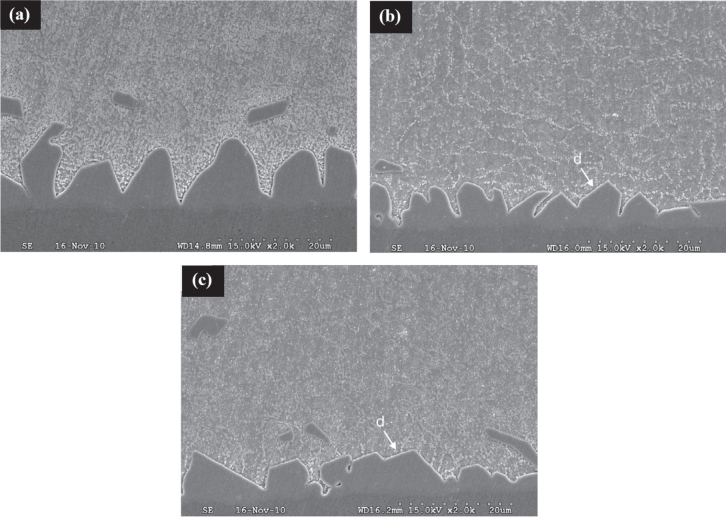
Cross sectional SEM micrographs of (a) SAC/Cu, (b) SAC–0.5Al_2_O_3_/Cu and (c) SAC–1.0Al_2_O_3_/Cu joint interfaces after eight cycles of reflowing. Adapted from Tsao *et al* [35], copyright 2013, with permission from Elsevier.

On the other hand, Tsao *et al* [35] reported that the growth of the Cu_6_Sn_5_ IMC layer was retarded when the SAC–(0.5–1.0 wt%)Al_2_O_3_/Cu soldered joints were subjected to multiple reflow cycles. The Cu_6_Sn_5_ IMC morphology changed from scallop-like to prism-like or faceted as shown in figures 10(b) and (c). The IMC thickness was thinner in the composite solder joint when compared to the SAC/Cu solder joint. The authors suggested that the adsorption effect of Al_2_O_3_ nanoparticles on the Cu_6_Sn_5_ IMC grains can effectively limit the dissolution of Cu atoms into the liquid solder. Hence, the grain ripening effect was reduced and the formation of the scallop-shaped Cu_6_Sn_5_ IMC was retarded. The Cu_6_Sn_5_ IMC became more faceted when the amount of Al_2_O_3_ nanoparticles added increased.

Based on the results presented above, we can see that Al_2_O_3_ nanoparticles can effectively retard the growth of the Cu_6_Sn_5_ IMC in general soldering condition.

#### 3.2.2. Titanium dioxide (TiO_2_)

The effects of the addition of titanium dioxide (TiO_2_) nanoparticles in lead-free solders have been well reported in the last decade. Lin *et al* [96] and Tsao *et al* [65, 97, 98] reported that the mechanical properties of the composite solder such as ultimate tensile strength, yield strength and microhardness increased when the TiO_2_ content increased from 0.25 to 1.0 wt%. These enhancements were contributed by the dispersion of refined IMC particles (such as Cu_6_Sn_5_ and Ag_3_Sn nanoparticles) and TiO_2_ nanoparticles in the solder matrix which acted as reinforcements in the solder and obstacles for solder dislocation. However, the increasing TiO_2_ content deteriorated the ductility of the composite solder. Furthermore, micropores were present in the bulk solder when the TiO_2_ content was 1.0 wt% and above due to the agglomeration and segregation of TiO_2_ nanoparticles. Shi *et al* [99] found that the creep resistance of the composite solder increased with increasing the TiO_2_ nanoparticle content up to 3.0 wt%.

In the SAC–TiO_2_/Cu solder joint, the growth of the Cu_6_Sn_5_ IMC at the interface was retarded due to the adsorption effect of TiO_2_ nanoparticles. Chang *et al* [100] explained that the Ag_3_Sn particles are larger than the TiO_2_ nanoparticles, and thus the smaller particles will adsorb on the larger particles. When the Ag_3_Sn particles grew during solidification and had the maximum adsorption of TiO_2_ nanoparticles, the surface energy of the Ag_3_Sn particles decreased which in turn, suppressed the growth and refined the Ag_3_Sn particles into nano-sizes. Similarly, the adsorption of Ag_3_Sn nanoparticles on Cu_6_Sn_5_ IMC grains at the joint interface retarded and refined the IMC grains. The authors observed that the IMC thickness decreased by approximately 50% when 0.5 wt% TiO_2_ nanoparticles were added to the SAC/Cu solder joint. Moreover, the morphology of the Cu_6_Sn_5_ IMC remained scallop-like instead of whisker-like during prolonged reflow times as the adsorption of nanoparticles reduced the ripening rate of the IMC grains.

The finding was in accordance with the work of Leong [101] and Tsao [8, 102]. Tsao [8] further investigated the IMC growth in the thermally aged SAC–0.5TiO_2_/Cu solder joints. Ag_3_Sn nanoparticles were found on top of aged Cu_6_Sn_5_ IMC grains and the activation energy of the IMC was overall higher than those in the SAC/Cu solder joint. Therefore, he suggested that the adsorption of Ag_3_Sn nanoparticles on Cu_6_Sn_5_ IMC grains not only reduces the grain’s surface energy, but also acts as a diffusion barrier which reduces the diffusion rate of the Sn atoms into the solder/Cu_6_Sn_5_ interface.

The suppression of Cu_6_Sn_5_ IMC growth also occured when the composite joints were subjected to multiple reflow cycles according to Gain *et al* [6]. However, the authors said that the suppression effect was due to the adsorption of TiO_2_ nanoparticles on the IMC grains rather than the Ag_3_Sn nanoparticles. The formation of Ag_3_Sn nanoparticles was not reported as well in the work of Tang *et al* [18]. Furthermore, the authors noticed that the suppression effect when adding 0.1 wt% TiO_2_ nanoparticles is better than adding over 0.1 wt%. This was due to the agglomeration and uneven distribution of the excessive nanoparticles, which reduced the effect of IMC growth suppression.

Therefore, we can conclude that TiO_2_ nanoparticles can effectively retard the growth of the Cu_6_Sn_5_ IMC in the overall situation. However, precautions should be taken to ensure an even distribution of these particles and prevent them from agglomerating.

#### 3.2.3. Zirconium dioxide (ZrO_2_)

Shen *et al* [103, 104] and Gain *et al* [105] reported that the addition of ZrO_2_ nanoparticles (1 to 2 wt%) in lead-free solder could reduce the size of the *β*-Sn grains and restrict the growth of Ag_3_Sn IMC particles present in the bulk solder due to the adsorption effect. Increasing amounts of addition would increase the refinement of the IMC and consequently increase the microhardness of the composite solder. Zhong *et al* [93] found that ZrO_2_-added solder has a higher yield strength and ultimate tensile strength (UTS) than ZrO_2_-free solder but the formation of pores was observed in the composite solder. Moreover, Shen and Chan [106] revealed that the addition of ZrO_2_ nanoparticles to the solder flux, which was applied between the SAC solder ball and Cu substrate, would reduce the wettability of molten SAC on the substrate. This is because ZrO_2_ nanoparticles do not react with or blend into the molten solder and accumulate on top of the Cu substrate, which in turn impedes the spreading of molten solder.

Gain *et al* [105] reported that the addition of ZrO_2_ nanoparticles in the SAC/Cu solder joint did not alter the composition and morphology of the Cu_6_Sn_5_ IMC, and the IMC thickness was slightly thinner than in the plain SAC/Cu solder joint. IMC suppression also occurred in the joint soldered at higher soldering temperatures and higher reflow times. The activation energy of the total IMC layers in the composite joint was 59.5 kJ mol^−1^, which is higher than those in the SAC/Cu joint, which is 53.2 kJ mol^−1^. This indicates that more energy was required to form the IMCs when ZrO_2_ nanoparticles were added to the solder.

In another research of Gain *et al* [107], ZrO_2_, TiO_2_ and Al_2_O_3_-added composite joints were subjected to thermal cycling. The IMC suppression of ZrO_2_ particles was rather weak compared to TiO_2_ and Al_2_O_3_. However, the suppression effect became more significant when the amount of addition increased to 3.0 wt%. The authors further investigated the performance of ZrO_2_ nanoparticles in thermally aged SAC/Cu solder joints. The growth of both Cu_6_Sn_5_ and Cu_3_Sn IMCs was suppressed but the effect was rather weak as well [108]. IMC suppression may be contributed by the adsorption of ZrO_2_ nanoparticles which reduce the growth rate of Cu_6_Sn_5_ IMC grains and slow down the diffusion of Sn atoms into the solder/Cu_6_Sn_5_ interface [105, 107].

### 3.3. Carbon nanotube

#### 3.3.1. Carbon nanotube (CNT)

Over the last decade, carbon nanotubes have been introduced into conventional solders by several researchers and the composite solders are found to have better mechanical and thermal properties. Nai *et al* [109, 110] reported that SAC–CNT composite solder has better wettability and is dimensionally more stable than plain SAC as the composite solder possesses a lower CTE. Increasing the amount of CNTs in the solder could increase yield strength and ultimate tensile strength of the composite solder but deteriorate its ductility. The findings correspond to the work of Kumar *et al* [81, 111, 112]. The authors further explained that the deterioration in solder ductility was attributed to the CNTs, which acted as crack nucleation sites. This was evidenced by the discovery of CNTs at the solder fractured surface. Excessive addition of CNTs would reduce the solder wettability as the interaction between solder and substrate elements could be severely blocked by excessive CNTs [113, 114].

**Figure 11.**
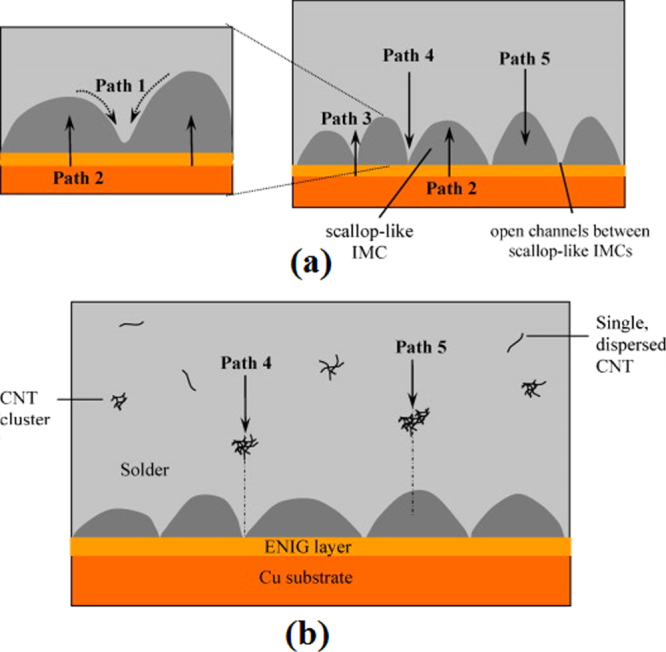
Schematic diagram of possible diffusion paths in a solder joint; (a) SAC and (b) SAC-CNT. Adapted from Nai *et al* [115], copyright 2009, with permission from Elsevier.

In general, for the addition of CNTs, they do not interact with the elements in the joint and thus the morphology of the IMC at the joint interface remains unchanged. The Cu_6_Sn_5_ IMC layer thickness in the as-soldered and thermally aged joints is suppressed with the addition of CNT but the effect was rather weaker than for the other composite solders mentioned earlier [114–116]. According to Nai *et al* [115], the CNT was in the form of a single-dispersed strand and a CNT cluster after being added into the solder (as shown in figure 11(b)). They became a diffusion barrier for Sn atoms in the solder matrix thus retarding the Cu_6_Sn_5_ IMC growth.

However, in the work of Ko *et al* [117], the efficiency of the suppression effect in the as-soldered and thermally aged joints was very much improved when the authors implemented a new composite solder fabrication method which is called surface impact mixing (SIM). Solder balls were used in this work instead of solder paste. CNTs were embedded on the solder surface through a ball milling process for up to 24 h which provided sufficient impact energy for the embedment. Through this method, CNTs were well-dispersed even in a dry state and did not agglomerate easily as they were embedded on the solder ball surface. Consequently, the IMC suppression effect of CNT improved greatly.

#### 3.3.2. Ni-coated carbon nanotube (Ni-CNT)

The great impact of CNTs on lead-free solder properties has drawn the attention of several researchers to further investigate possible ways of improving its IMC suppression efficiency. Han *et al* [118] identified that the inhomogeneous dispersion of CNTs in the solder matrix and insufficient bonding between the CNTs and the matrix elements are the limiting factors of suppression efficiency. Surface coating on the nanotubes is one of the feasible ways to promote bonding between CNTs and solder elements, and nickel (Ni) has been chosen as the coating material as it can form stable phases, Ni_3_Sn_4_, with Sn from the solder. In addition, a continuous coating can be formed on the CNTs with a significant binding energy [119, 120]. In the studies of Han *et al*, the addition of 0.05 wt% of Ni-CNTs into SAC solder has shown the most pronounced enhancement in terms of solder properties. The composite solder has higher strength, lower CTE [118], better creep performance [121], and better corrosion resistance [122]. Yang *et al* [123] reported that Ni-CNT added solder joint has better electromigration resistance as the atomic diffusion induced by electromigration in the solder is retarded by Ni-coated CNT single strands or clusters.

When Ni-CNTs was added into a SAC solder joint, the Ni atoms coated on CNTs behaved similar to the Ni particles addition. A (Cu,Ni)_6_Sn_5_ IMC was formed and it was relatively thicker than an ordinary Cu_6_Sn_5_ IMC [123, 124]. However, the growth of the (Cu,Ni)_6_Sn_5_ IMC was suppressed significantly when the joint was subjected to an electromigration test, in which a current passed through the solder joint and caused electrons to collide with atoms in the solder to conduct electricity [123]. This would induce the atomic diffusion of Sn and Cu atoms in a plain SAC solder joint. But in the case of a Ni-CNT added solder joint, the electrons would go through the Ni-CNTs rather than colliding with the atoms due to Ni-CNTs providing better electrical conductivity. Hence, the atomic diffusion of Sn and Cu atoms was reduced and the growth of the (Cu,Ni)_6_Sn_5_ IMC was retarded.

In the research of Han *et al* [24, 125], the effect of Ni-CNTs on the IMC growth after soldering, thermal aging and thermal cycling was examined. The growth of the (Cu,Ni)_6_Sn_5_ IMC layer was suppressed after thermal aging and the thermal cycling test. The authors explained that the suppression effect was attributed to (1) the reaction between Ni atoms from the Ni-CNTs with Sn atoms to form a Ni_3_Sn_4_ IMC in the solder matrix, (2) the Ni_3_Sn_4_ IMC and Ni-CNTs in the solder matrix acted as a diffusion barrier for Sn atoms to reach solder/(Cu,Ni)_6_Sn_5_ interface, and (3) the lower CTE of the composite solder which can reduce the atomic diffusion of Sn and Cu atoms induced by the relief of compressive and tensile stresses in a thermally cycled and thermally aged joint.

In summary, surface coated CNTs demonstrated better IMC suppression efficiency compare to ordinary CNTs. 0.05 wt% is the optimized amount of addition reported so far, based on the literature.

## 4. Summary and future directions

Literature suggests that addition of a small amount of nanoparticles significantly affects the formation and growth of IMCs in a solder joint. Three types of nanoparticles have been reviewed in this paper: metallic, ceramic and carbon nanotubes. We categorize the nanoparticle elements into two general groups: (1) elements that dissolve in Cu–Sn IMCs and (2) elements that doenot dissolve in Cu–Sn IMCs. Ni, Co and Ni-CNT are considered as group (1) elements as they dissolved into Cu–Sn IMCs and formed (Cu,Ni)_6_Sn_5_ and (Cu,Co)_6_Sn_5_ respectively. These new IMCs are more thermodynamically stable than the Cu_6_Sn_5_ IMC and would not easily decompose to Cu_3_Sn especially during thermal aging and thermal cycling. The group (2) elements consist of Al, Mo, Al_2_O_3_, TiO_2_, ZrO_2_ and CNTs. This group of elements could reduce the growth of Cu–Sn IMCs in a solder joint by the adsorption effect. However, Zn is considered as an exception as it does not behave like the other elements from these two groups. Instead, Zn reacted with Cu directly and formed a Cu_5_Zn_8_ IMC layer on top of the Cu_6_Sn_5_ IMC when the Zn content is above 1.0 wt%.

From the above literature, the addition amount is crucial in regulating the growth of Cu–Sn IMCs of a solder joint. When excessive amounts of nanoparticles are added into the joint, the spreadability of the molten solder on the substrate would be reduced due to the higher viscosity. Additional brittle IMCs might be formed in the solder joint. Furthermore, excessive nanoparticles would agglomerate and result in the formation of micropores at the joint interface. The effect of each element on the growth of Cu–Sn IMCs during different thermal conditions has been summarized in table 4 and we can basically conclude that the effective amount of addition of any of these elements is not more than 3.0 wt%.

**Table 4.** Effect of nanoparticle composition on the growth of Cu–Sn.

		Change of IMC thickness compare with SAC solder joint								
		As-soldered		Prolonged soldered		Thermally aged		Thermally cycled		
Nano-particle	wt%	Cu_6_Sn_5_	Cu_3_Sn	Cu_6_Sn_5_	Cu_3_Sn	Cu_6_Sn_5_	Cu_3_Sn	Cu_6_Sn_5_	Cu_3_Sn	Reference
	0.5–3.0	↑	—	—	↑↑	↓↓	—	—	[17, 53, 70]
Ni	0.02–0.6	↑	—	↓ (ovr)		—	—	—	—	[18]
	0.5	—	—	—	↓	↑	—	[69]
Al	1.0–3.0	↓ (ovr)		—	—	—	↓ (ovr)	↓ (ovr)		[76]
Co	0.5–1.5	↑	—	—	—	↑↑	↓↓	—	—	[48]
Mo	1.0–2.0	↓	—	↓↓ (ovr)		—	—	↓↓	↓	[7, 83]
	1.0–2.0	↓↓	—	—	—	—	—	↓↓	↓↓	[87]
Zn	0.5–1.5	↓	—	—	—	↓↓	↓↓	—	—	[30]
	0.5	↓	—	↓(ovr)	—	—		[95]
Al_2_O_3_	0.25–1.0	↓	—	—		—	—	↓↓ (ovr)		[35]
	0.5	↓↓	—	↓↓ (ovr)	—	—	[100, 101]
	0.5	↓	—	—	↓ (ovr)	—	[8]
TiO_2_	1.0	↓		—	—	↓ (ovr)	[6]
	0.02–0.06	↓		↓ (ovr)		—		—		[18]
	1.0	↓ (ovr)	↓ (ovr)	↓	↓	—	[105, 108]
ZrO_2_	3.0	↓ (ovr)	—	—		—		↓ (ovr)		[107]
CNT	0.01–1.0	↓ (ovr)		—	—	↓ (ovr)		—	—	[114–116]
Ni–CNT	0.01	↓ (ovr)	—	—	↓ (ovr)	↓ (ovr)	[125]

Notes: ↓ = slightly reduced ↓↓ = significantly reduced ↓ (ovr) = reduced completely ↑ = slightly increased ↑↑ = significantly increased

From table 4, we can see that elements from group (1) have the tendency to promote Cu_6_Sn_5_ IMC and suppress Cu_3_Sn IMC growth at the same time. This implies that better control of the process parameters is required while using these elements as their suppressing effect might be very dependent on parameters such as temperature and time. In contrast, elements from group (2) which are considered as non-reacting elements, have a stable suppression effect on both IMCs regardless of temperature and time. Amongst all the nanoparticles reviewed in the paper, Mo and Zn nanoparticles are the elements that have the most remarkable effect on suppressing the IMC’s growth under all thermal conditions discussed.

However, the reliability of a composite solder joint cannot be judged solely based on its effectiveness in regulating the IMC’s growth. In the studies of Lin and Chuang [86], Gain *et al* [107] and Lee *et al* [63], the addition of Zn, ZrO_2_ and Co particles was reported to be effective in reducing the IMC’s growth. However, different observations on the joint shear strength and fracture mode were observed in these studies. Lin and Chuang [86] reported that an addition of 0.5 wt% Zn degraded the shear strength of a SAC/Cu solder joint, but the shear fracture mode remained ductile. Gain *et al* [107] found that the addition of up to 3 wt% ZrO_2_ nanoparticles improved the joint shear strength, and the shear fracture mode changed from brittle to ductile fracture after the addition. In the work of Lee *et al* [63], addition of 1 wt% Co improved the SA/Cu joint shear strength but the joint strength with 2 wt% Co addition was the same as the plain SA/Cu joint, and the fracture mode changed from ductile to brittle after the addition of Co particles. Therefore, based on these studies, it can be clearly seen that the joint shear strength and ductility should be taken into consideration in the evaluation of joint reliability, since the addition of nanoparticles has demonstrated different effects on these two mechanical properties, even though they have been proven to have suppressing effects on the IMC’s growth. Further studies need to be conducted in the future to address this issue. Thus, in our opinion, it should remain a subject of further investigation in the field of electronic packaging processing.

There has been some recent development in the fabrication of new composite solders by adding new ceramic and polymeric nanoparticles such as ZnO [126], Y_2_O_3_ [127], SiC [19, 128], Si_3_N_4_ [129] and polyhedral oligomeric silsesquioxanes (POSS) [130]. It is reported that these new composite solders show promising enhancements on solder and solder joint properties. Therefore, it is foreseen that non-reacting and non-metallic nanoparticles would continue to attract interest as potential candidates for fabrication of nanocomposite lead-free solder in the application of electronic packaging.

## References

[C1] Kim K S, Huh S H, Suganuma K (2003). J. Alloys Compd..

[C2] Chan Y C, So A C K, Lai J K L (1998). Mater. Sci. Eng. B.

[C3] Hodúlová E, Palcut M, Lechovič E, Šimeková B, Ulrich K (2011). J. Alloys Compd..

[C4] Liu L, Zhou W, Li B, Wu P (2009). J. Alloys Compd..

[C5] Tsukamoto H, Nishimura T, Suenaga S, Nogita K (2010). Mater. Sci. Eng. B.

[C6] Gain A K, Chan Y C, Yung W K C (2011). Microelectron. Reliab..

[C7] Haseeb A S M A, Arafat M M, Johan M R (2012). Mater. Charact..

[C8] Tsao L C (2011). J. Alloys Compd..

[C9] Cheng F, Gao F, Nishikawa H, Takemoto T (2009). J. Alloys Compd..

[C10] Wu C M L, Yu D Q, Law C M T, Wang L (2004). Mater. Sci. Eng. R.

[C11] Marques V M F, Johnston C, Grant P S (2013). Acta Mater..

[C12] Li J F, Agyakwa P A, Johnson C M (2012). J. Alloys Compd..

[C13] Dariavach N, Callahan P, Liang J, Fournelle R (2006). J. Elec. Mater..

[C14] Shen J, Chan Y C (2009). Microelectron. Reliab..

[C15] Zeng G, McDonald S, Nogita K (2012). Microelectron. Reliab..

[C16] Dutta I, Majumdar B S, Pan D, Horton W S, Wright W, Wang Z X (2004). J. Elec. Mater..

[C17] Tay S L, Haseeb A S M A, Johan M R, Munroe P R, Quadir M Z (2013). Intermetallics.

[C18] Tang Y, Li G Y, Pan Y C (2013). J. Alloys Compd..

[C19] El-Daly A A, Fawzy A, Mansour S F, Younis M J (2013). Mater. Sci. Eng. A.

[C20] Mavoori H, Jin S (1998). J. Elec. Mater..

[C21] Mavoori H, Jin S (2000). JOM.

[C22] Chuang T H, Tsao L C, Chung C-H, Chang S Y (2012). Materials & Design.

[C23] Amagai M (2008). Microelectron. Reliab..

[C24] Han Y D, Jing H Y, Nai S M L, Xu L Y, Tan C M, Wei J (2012). Intermetallics.

[C25] Fouzder T, Shafiq I, Chan Y C, Sharif A, Yung W K C (2011). J. Alloys Compd..

[C26] Zhang L, Xue S B, Zeng G, Gao L L, Ye H (2012). J. Alloys Compd..

[C27] Rizvi M J, Chan Y C, Bailey C, Lu H, Islam M N (2006). J. Alloys Compd..

[C28] Yang M, Li M, Wang C (2012). Intermetallics.

[C29] Gong J, Liu C, Conway P P, Silberschmidt V V (2009). Scr. Mater..

[C30] Kotadia H R, Mokhtari O, Clode M P, Green M A, Mannan S H (2012). J. Alloys Compd..

[C31] Hirose A, Yanagawa H, Ide E, Kobayashi K F (2004). Sci. Technol. Adv. Mater..

[C32] Laurila T, Vuorinen V, Paulasto-Kröckel M (2010). Mater. Sci. Eng. R.

[C33] Tu K-N (2007). Solder Joint Technology.

[C34] Suh J O, Tu K N, Lutsenko G V, Gusak A M (2008). Acta Mater..

[C35] Tsao L C, Wu R W, Cheng T-H, Fan K-H, Chen R S (2013). Materials & Design.

[C36] Gong J, Liu C, Conway P P, Silberschmidt V V (2008). Acta Mater..

[C37] Gagliano R, Fine M (2001). JOM.

[C38] Park M S, Arróyave R (2012). Acta Mater..

[C39] Zhang Z, Li M, Wang C (2013). Intermetallics.

[C40] Qu L, Zhao N, Zhao H J, Huang M L, Ma H T (2014). Scr. Mater..

[C41] Ely D R, Edwin García R, Thommes M (2014). Powder Technol..

[C42] Perez M (2005). Scr. Mater..

[C43] Baldan A (2002). J. Mater. Sci..

[C44] Liu W, Tian Y, Wang C, Wang X, Liu R (2012). Mater. Lett..

[C45] Tian Y, Zhang R, Hang C, Niu L, Wang C (2014). Mater. Charact..

[C46] Pang J H L, Low T H, Xiong B S, Luhua X, Neo C C (2004). Thin Solid Films.

[C47] Mookam N, Kanlayasiri K (2012). J. Mater. Sci. Technol..

[C48] Haseeb A S M A, Leng T S (2011). Intermetallics.

[C49] Wang Y W, Lin Y W, Tu C T, Kao C R (2009). J. Alloys Compd..

[C50] Shen J, Zhao M, He P, Pu Y (2013). J. Alloys Compd..

[C51] Peng W, Monlevade E, Marques M E (2007). Microelectron. Reliab..

[C52] Tz-Cheng C, Kejun Z, Stierman R, Edwards D, Ano K (2004).

[C53] Liu P, Yao P, Liu J (2009). J. Alloys Compd..

[C54] Tang W-M, He A-Q, Liu Q, Ivey D G (2010). Trans. Nonferrous Metals Soc. China.

[C55] Liu C-Y, Lai C-H, Wang M-C, Hon M-H (2006). J. Cryst. Growth.

[C56] Yoon J-W, Noh B-I, Kim B-K, Shur C-C, Jung S-B (2009). J. Alloys Compd..

[C57] Kim Y, Roh H-R, Kim S, Kim Y-H (2010). J. Elec. Mater..

[C58] Huang N, Hu A, Li M (2013). Mater. Lett..

[C59] Teo J W R, Sun Y F (2008). Acta Mater..

[C60] Han Y D, Jing H Y, Nai S M L, Xu L Y, Tan C M, Wei J (2012). Intermetallics.

[C61] Lihua Q, Jihua H, Jing N, Long Y, Yaorong F, Xingke Z, Hua Z (2009).

[C62] Zeng G, Xue S, Zhang L, Gao L, Dai W, Luo J (2010). J. Mater. Sci., Mater. Electron..

[C63] Lee J-S, Chu K-M, Patzelt R, Manessis D, Ostmann A, Jeon D Y (2008). Microelectron. Eng..

[C64] Mohankumar K, Tay A A O (2004). Nano-particle reinforced solders for fine pitch applications. EPTC 2004: Proc. 6th Electronics Packaging Technology Conference.

[C65] Tsao L C, Huang C H, Chung C H, Chen R S (2012). Mater. Sci. Eng. A.

[C66] Wilde G, Perepezko J H (2000). Mater. Sci. Eng. A.

[C67] Niranjani V L, Chandra Rao B S S, Sarkar R, Kamat S V (2012). J. Alloys Compd..

[C68] Yao P, Liu P, Liu J (2008). J. Alloys Compd..

[C69] Gain A K, Chan Y C (2012). Intermetallics.

[C70] Lee H T, Lee Y H (2006). Mater. Sci. Eng. A.

[C71] Yoon J-W, Kim S-W, Jung S-B (2005). J. Alloys Compd..

[C72] Laurila T, Vuorinen V, Kivilahti J K (2005). Mater. Sci. Eng. R.

[C73] Gao F, Takemoto T, Nishikawa H (2006). Mater. Sci. Eng. A.

[C74] Shnawah D-A, Sabri M, Badruddin I, Said S, Che F (2012). J. Mater. Sci., Mater. Electron..

[C75] Kotadia H R, Mokhtari O, Bottrill M, Clode M P, Green M A, Mannan S H (2010). J. Elec. Mater..

[C76] Gain A K, Fouzder T, Chan Y C, Sharif A, Wong N B, Yung W K C (2010). J. Alloys Compd..

[C77] Anderson I E, Harringa J L (2006). J. Elec. Mater..

[C78] Cheng F J, Nishikawa H, Takemoto T (2008). Mater. Trans..

[C79] Gao F, Qu J, Takemoto T (2010). J. Elec. Mater..

[C80] Anderson I E, Foley J C, Cook B A, Harringa J, Terpstra R L, Unal O (2001). J. Elec. Mater..

[C81] Kumar K M, Kripesh V, Tay A A O (2006).

[C82] Chandra Rao B S S, Mohan Kumar K, Zeng K Y, Tay A A O, Kripesh V (2009). Effect of strain rate and temperature on tensile flow behavior of SnAgCu nanocomposite solders. EPTC ’09: 11th Electronics Packaging Technology Conference, 2009.

[C83] Arafat M M, Haseeb A, Johan M R (2011). Solder. Surf. Mount Technol..

[C84] Song H Y, Zhu Q S, Wang Z G, Shang J K, Lu M (2010). Mater. Sci. Eng. A.

[C85] El-Daly A A, El-Taher A M (2013). Materials & Design.

[C86] Lin H-J, Chuang T-H (2010). J. Alloys Compd..

[C87] Chan Y H, Arafat M M, Haseeb A (2013). Solder. Surf. Mount Technol..

[C88] Hao H, Tian J, Shi Y W, Lei Y P, Xia Z D (2007). J. Elec. Mater..

[C89] Prasad L C, Mikula A (2006). Physica B.

[C90] Yahya I, Ab Ghani N A, Abiddin N N Z, Abd Hamid H, Mayappan R (2013). Adv. Mater. Res..

[C91] Tsao L C, Chang S Y, Lee C I, Sun W H, Huang C H (2010). Mater. Des..

[C92] Zhong X L, Gupta M (2008). J. Phys. D: Appl. Phys..

[C93] Zhong X L, Gupta M (2008). Effect of type of reinforcement at nanolength scale on the tensile properties of Sn-0.7Cu solder alloy. EPTC 2008: 10th Electronics Packaging Technology Conference, 2008.

[C94] Chuang T H, Wu M W, Chang S Y, Ping S F, Tsao L C (2011). J. Mater. Sci., Mater. Electron..

[C95] Chang S Y, Tsao L C, Wu M W, Chen C W (2012). J. Mater. Sci., Mater. Electron..

[C96] Lin D C, Wang G X, Srivatsan T S, Al-Hajri M, Petraroli M (2003). Mater. Lett..

[C97] Tsao L C (2011). Mater. Sci. Eng. A.

[C98] Tsao L C, Chang S Y (2010). Mater. Des..

[C99] Shi Y W, Liu J P, Xia Z D, Lei Y P, Guo F, Li X Y (2008). J. Mater. Sci., Mater. Electron..

[C100] Chang S Y, Jain C C, Chuang T H, Feng L P, Tsao L C (2011). Mater. Des..

[C101] Leong J C, Tsao L C, Fang C J, Chu C P (2011). J. Mater. Sci., Mater. Electron..

[C102] Tsao L C (2011). J. Alloys Compd..

[C103] Shen J, Liu Y C, Wang D J, Gao H X (2006). J. Mater. Sci. Technol..

[C104] Shen J, Liu Y C, Han Y J, Tian Y M, Gao H X (2006). J. Elec. Mater..

[C105] Gain A K, Fouzder T, Chan Y C, Yung W K C (2011). J. Alloys Compd..

[C106] Shen J, Chan Y C (2009). J. Alloys Compd..

[C107] Gain A K, Chan Y C, Yung W K C (2011). Microelectron. Reliab..

[C108] Gain A K, Chan Y C (2014). Microelectron. Reliab..

[C109] Nai S M L, Wei J, Gupta M (2006). Thin Solid Films.

[C110] Nai S M L, Wei J, Gupta M (2006). Mater. Sci. Eng. A.

[C111] Kumar K M, Kripesh V, Tay A A O (2008). J. Alloys Compd..

[C112] Kumar K M, Kripesh V, Shen L, Tay A A O (2006). Thin Solid Films.

[C113] Jakubowska M, Bukat K, Koscielski M, Mlozniak A, Niedzwiedz W, Sloma M, Sitek J (2010).

[C114] Huang H Y, Yang C W, Pan S Z (2013). Sci. Eng. Compos. Mater..

[C115] Nai S M L, Wei J, Gupta M (2009). J. Alloys Compd..

[C116] Xu S, Chan Y C, Zhang K, Yung K C (2014). J. Alloys Compd..

[C117] Ko Y-K, Kwon S-H, Lee Y-K, Kim J-K, Lee C-W, Yoo S (2014). J. Alloys Compd..

[C118] Han Y D, Nai S M L, Jing H Y, Xu L Y, Tan C M, Wei J (2011). J. Mater. Sci., Mater. Electron..

[C119] Durgun E, Dag S, Bagci V M K, Gülseren O, Yildirim T, Ciraci S (2003). Phys. Rev. B.

[C120] Yang S H, Shin W H, Lee J W, Kim S Y, Woo S I, Kang J K (2006). J. Phys. Chem. B.

[C121] Han Y D, Jing H Y, Nai S M L, Xu L Y, Tan C M, Wei J (2012). J. Mater. Sci., Mater. Electron..

[C122] Han Y D, Chen L, Jing H Y, Nai S M L, Wei J, Xu L Y (2013). J. Elec. Mater..

[C123] Yang Z, Zhou W, Wu P (2013). J. Alloys Compd..

[C124] Yang Z, Zhou W, Wu P (2014). Mater. Sci. Eng. A.

[C125] Han Y D, Jing H Y, Nai S M L, Xu L Y, Tan C M, Wei J (2012). J. Elec. Mater..

[C126] Fawzy A, Fayek S A, Sobhy M, Nassr E, Mousa M M, Saad G (2014). Mater. Sci. Eng. A.

[C127] Yang L M, Zhang Z F (2013). Effects of Y2O3. J. Elec. Mater..

[C128] El-Daly A A, Fawzy A, Mansour S F, Younis M J (2013). J. Mater. Sci., Mater. Electron..

[C129] Mohd Salleh M A A, Bakri A M M A, Kamarudin H, Bnhussain M, Zan Hazizi M H, Somidin F (2011). Physics Procedia.

[C130] Shen J, Tang Q, Pu Y, Zhai D, Cao Z, Chen J (2013). J. Mat. Sci., Mat. Electron..

